# Identification of cell-type-specific mutations in nodal T-cell lymphomas

**DOI:** 10.1038/bcj.2016.122

**Published:** 2017-01-06

**Authors:** T B Nguyen, M Sakata-Yanagimoto, Y Asabe, D Matsubara, J Kano, K Yoshida, Y Shiraishi, K Chiba, H Tanaka, S Miyano, K Izutsu, N Nakamura, K Takeuchi, H Miyoshi, K Ohshima, T Minowa, S Ogawa, M Noguchi, S Chiba

**Affiliations:** 1 Department of Hematology, Graduate School of Comprehensive Human Sciences, University of Tsukuba, Tsukuba, Ibaraki, Japan; 2 Department of Hematology, Faculty of Medicine, University of Medicine and Pharmacy, Ho Chi Minh City, Vietnam; 3 Stem Cell Transplantation Zone, Blood Transfusion Hematology Hospital, Ho Chi Minh City, Vietnam; 4 Department of Hematology, Faculty of Medicine, University of Tsukuba, Tsukuba, Ibaraki, Japan; 5 Department of Hematology, University of Tsukuba Hospital, Tsukuba, Ibaraki, Japan; 6 Department of Integrative Pathology, Jichii Medical University, Shimotsuke, Tochigi, Japan; 7 Department of Pathology, Faculty of Medicine, University of Tsukuba, Tsukuba, Ibaraki, Japan; 8 Department of Pathology and Tumor Biology, Graduate School of Medicine, Kyoto University, Kyoto, Japan; 9 Human Genome Center, Institute of Medical Science, University of Tokyo, Tokyo, Japan; 10 Department of Hematology, Toranomon Hospital, Tokyo, Japan; 11 Okinaka Memorial Institute for Medical Research, Tokyo, Japan; 12 Department of Pathology, Tokai University School of Medicine, Isehara, Kanagawa, Japan; 13 Pathology Project for Molecular Targets, The Cancer Institute, Japanese Foundation for Cancer Research, Tokyo, Japan; 14 Department of Pathology, Kurume University, Kurume, Fukuoka, Japan; 15 Nanotechnology Innovation Station, National Institute for Materials Science, Tsukuba, Ibaraki, Japan

## Abstract

Recent genetic analysis has identified frequent mutations in *ten-eleven translocation 2* (*TET2*), *DNA methyltransferase 3A* (*DNMT3A*), *isocitrate dehydrogenase 2* (*IDH2*) and *ras homolog family member A* (*RHOA*) in nodal T-cell lymphomas, including angioimmunoblastic T-cell lymphoma and peripheral T-cell lymphoma, not otherwise specified. We examined the distribution of mutations in these subtypes of mature T-/natural killer cell neoplasms to determine their clonal architecture. Targeted sequencing was performed for 71 genes in tumor-derived DNA of 87 cases. The mutations were then analyzed in a programmed death-1 (PD1)-positive population enriched with tumor cells and CD20-positive B cells purified by laser microdissection from 19 cases. *TET2* and *DNMT3A* mutations were identified in both the PD1+ cells and the CD20+ cells in 15/16 and 4/7 cases, respectively. All the *RHOA* and *IDH2* mutations were confined to the PD1+ cells, indicating that some, including *RHOA* and *IDH2* mutations, being specific events in tumor cells. Notably, we found that all *NOTCH1* mutations were detected only in the CD20+ cells. In conclusion, we identified both B- as well as T-cell-specific mutations, and mutations common to both T and B cells. These findings indicate the expansion of a clone after multistep and multilineal acquisition of gene mutations.

## Introduction

Nodal T-cell lymphomas are subtypes of mature T-/natural killer-cell neoplasms, including angioimmunoblastic T-cell lymphoma (AITL); nodal peripheral T-cell lymphoma (PTCL) with T follicular helper (TFH) phenotype; peripheral T-cell lymphoma, not otherwise specified (PTCL-NOS), and follicular T-cell lymphoma. Among them, AITL is a distinct subtype, accounting for 16.0–28.7% of all mature T-/natural killer-cell neoplasms.^1, 2, 3^ AITL is characterized by specific clinical features, including generalized lymphadenopathy, high fever, skin rash and autoimmune-like manifestations. AITL tumor cells share characteristics with TFH cells, expressing B-cell lymphoma protein 6, a transcription factor; C-C motif chemokine receptor 5, a chemokine receptor; C-X-C motif ligand 13, a chemokine; and programmed death-1 (PD1), a member of the CD28 costimulatory membrane receptor family.^4, 5^ AITL tissues display prominent infiltration of inflammatory cells, follicular dendritic cell meshwork formation and branching vascular structures. Some nodal T-cell lymphomas exhibit several features reminiscent of AITL, although they do not show the typical morphology of AITL (nodal PTCL with TFH phenotype).^6, 7^ The massive infiltration of inflammatory cells in AITL has been explained by cytokines and chemokines being released from TFH-like tumor cells.^4^

Recurrent gene mutations have been identified in nodal T-cell lymphomas, including those in *ten-eleven translocation 2* (*TET2*) in 20–83%, *isocitrate dehydrogenase 2* (*IDH2*) in 0–45%, and *ras homolog family member A* (*RHOA*) in 17–71%, depending on the subtypes and *DNA methyltransferase 3A* (*DNMT3A*) in approximately 30%, independent of the subtypes.^8, 9, 10, 11, 12, 13^ Mutations in *TET2* encoding a methylcytosine dioxygenase and those in *DNMT3A* encoding a DNA methyltransferase presumably result in epigenetic abnormalities in nodal T-cell lymphomas. *IDH2* mutations also affect epigenetic modifications by inhibiting TET and histone demethylation enzymes through production of 2-hydroxyglutarate.^14^ Mutations in *RHOA* encoding a small GTPase are almost always located at the hotspot site, resulting in conversion from glycine to valine at the seventeenth position of the RHOA protein (G17V *RHOA* mutation). The G17V RHOA mutants could not be converted to an active GTP-bound form, although the downstream signaling of the G17V RHOA mutants in nodal T-cell lymphomas development has yet to be clarified.^8, 9, 13^

*TET2* and *DNMT3A* mutations are proposed to arise in hematopoietic stem/progenitors upstream of T-lineage commitment. This hypothesis is based on the fact that identical *TET2* and *DNMT3A* mutations were found in both tumor tissues and apparently normal blood cells in some AITL and PTCL-NOS patients.^8, 10, 15, 16, 17^ In contrast, the origins of the G17V *RHOA* mutation remain to be elucidated: it may be a tumor-specific event, considering that the allele frequencies of G17V *RHOA* mutations were lower than those of *TET2* mutations and that G17V *RHOA* mutations were found in only CD4+T lymphocytes in 1 AITL and 1 PTCL-NOS case.^8^

Here we describe the clonal architecture of nodal T-cell lymphomas by determining the distribution of mutations in enriched tumor cells and infiltrated B cells.

## Materials and methods

### Patients and samples

Samples, obtained from 87 patients (Supplementary Table S1) with AITL (*n*=48), nodal PTCL with TFH phenotype (*n*=5) and either of PTCL-NOS or of nodal PTCL with TFH phenotype (PTCL-NOS/nodal PTCL with TFH phenotype, *n*=34),^5^ were used after approval was obtained from the local ethics committees of all the participating institutes.

Genomic DNA was extracted from 56 fresh frozen samples using the Puregene DNA Blood Kit (Qiagen, Hilden, Germany) and 31 periodate-lysine-paraformaldehyde (PLP)-fixed frozen samples using the QIAamp DNA FFPE Tissue Kit (Qiagen).

### Targeted sequencing

Targeted sequencing was performed for 71 genes, which are listed in Supplementary Table S2. Sixty-one of the genes were previously screened by whole-exome sequencing,^8^ while 6 were the family genes of those whose mutations were identified by the whole-exome sequencing. The other four genes were deemed susceptible to mutations in PTCLs on the basis of the mutational profiles of other lymphoid malignancies.^18, 19, 20, 21, 22, 23^ All the exons of the selected genes were captured by use of a SureSelect Target Enrichment Kit (Agilent, Santa Clara, CA, USA) and then massively sequenced using HiSeq2000 (Illumina, Santa Clara, CA, USA). For each sample, all the sequencing reads were aligned to hg19 using BWA version 0.5.8 with default parameters. After all the duplicated reads and the low-quality reads and bases were removed, the allele frequencies of single-nucleotide variants and indels at each genomic position were calculated by enumerating the relevant reads using SAMtools (http://www.htslib.org). Initially, all the variants showing allele frequencies >0.02 were extracted and annotated using ANNOVAR^24, 25^ for further consideration, if they were found in >6 reads of >10 total reads and appeared in both the positive- and the negative-strand reads. All synonymous variants and known single-nucleotide polymorphisms in public and private databases, including dbSNP131, the 1000 genomes project as of 2012/05/21 and our in-house database, were removed. To exclude germline variants, nonsynonymous variants were excluded when the allele frequencies were from 0.45 to 0.55. Candidate mutations were validated by amplicon-based deep sequencing using Ion PGM (Life Technologies, Carlsbad, CA, USA) and/or Sanger sequencing (see below).

In the cohort of 87 cases, 79 were analyzed for *RHOA*, *TET2*, *DNMT3A* and *IDH2* mutations, and the results of this analysis were described in the previous paper.^7^ Now, eight were new cases. We re-analyzed all the 87 samples for targeted sequencing of 71 genes.

### Amplicon-based sequencing

The libraries were prepared using the Ion Plus Fragment Library Kit according to the protocol for preparing short amplicon libraries (Life Technologies). Briefly, PCR amplicons were ligated to the barcode adapters and P1 adapters and then amplified. The amplified libraries were quantitated by quantitative PCR with the Ion Library Quantitation Kit according to the manufacturer's instructions (Life Technologies). The libraries were then subjected to deep sequencing on the Ion Torrent PGM platform according to the standard protocol for 300 base-pair single-end reads (Life Technologies). The data were analyzed using Variant Caller 3.4 (Life Technologies).

### Immunohistochemistry

PLP-fixed frozen samples were cut in a cryostat at −22 °C into 5-μm sections and mounted on PEN-Membrane slides (Leica, Wetzlar, Germany). The tissue sections were stained with mouse anti-human PD1 (NAT105 ab52587, Abcam, Cambridge, UK) and anti-human CD20cy (clone L26, Dako, Michigan, MI, USA) antibodies, diluted 1:2000 and 1:1000, respectively, and detected by use of the Envision^+^ Dual Link System-HRP (Dako). The tissue sections were then counterstained with hematoxylin (Mayer's hematoxylin, Muto Pure Chemical, Tokyo, Japan) for 20 s at room temperature. After staining, tissue sections were dehydrated with ethanol and dried at room temperature before laser microdissection (LMD).

### LMD, DNA extraction and PCR

Nineteen of the 87 cases (13 AITL, 1 nodal PTCL with TFH phenotype and PTCL-NOS/nodal PTCL with TFH phenotype) were analyzed by LMD, which was performed using LMD7000 (Leica). The cells being positive for either PD1 or CD20 were dissected and collected into 0.2-ml PCR tubes (Takara, Shiga, Japan) containing 20 μl of distilled water. Stained cells at approximately 100 000 μm^2^ were dissected and collected for each sample. Genomic DNA was extracted using the QIAamp DNA PFFE Tissue Kit (Qiagen) following the manufacturer's protocol. Then 1 μl of DNA was used for PCR under the following conditions: 95 °C for 15 min, 60 °C for 4 min, 72 °C for 4 min, 35 to 40 cycles at 95 °C for 1 min, 60 °C for 1 min, 72 °C for 1 min, and 72 °C for 10 min using the AmpliTaq Gold 360 Kit (Applied Biosystems, Foster City, CA, USA) with each primer set (Supplementary Table S6). PCR amplicons were used for amplicon-based sequencing and Sanger sequencing.

### IgH gene rearrangement analysis and subcloning of the PCR product

Multiplex PCR assays were used to detect the clonality of B cells according to the European BIOMED-2 collaborative study.^26^ PCR products migrating at the expected size were extracted and sequenced using the Sanger method. Subcloning was performed if the Sanger sequencing indicated a polyclonal background by use of the pGEM-T Easy Vector System I (Promega, Madison, WI, USA). At least 12 colonies were picked up and sequenced to confirm the clonal expansion. The sequence results were analyzed using the IMGT tools^27^ and aligned to the closest match with the germline IGHV segment. Sequencing results with a germline identity of <98% were regarded as mutated and vice versa according to previous study.^28^

## Results

### Novel recurrent mutations in nodal T-cell lymphomas

**Figure 1:**
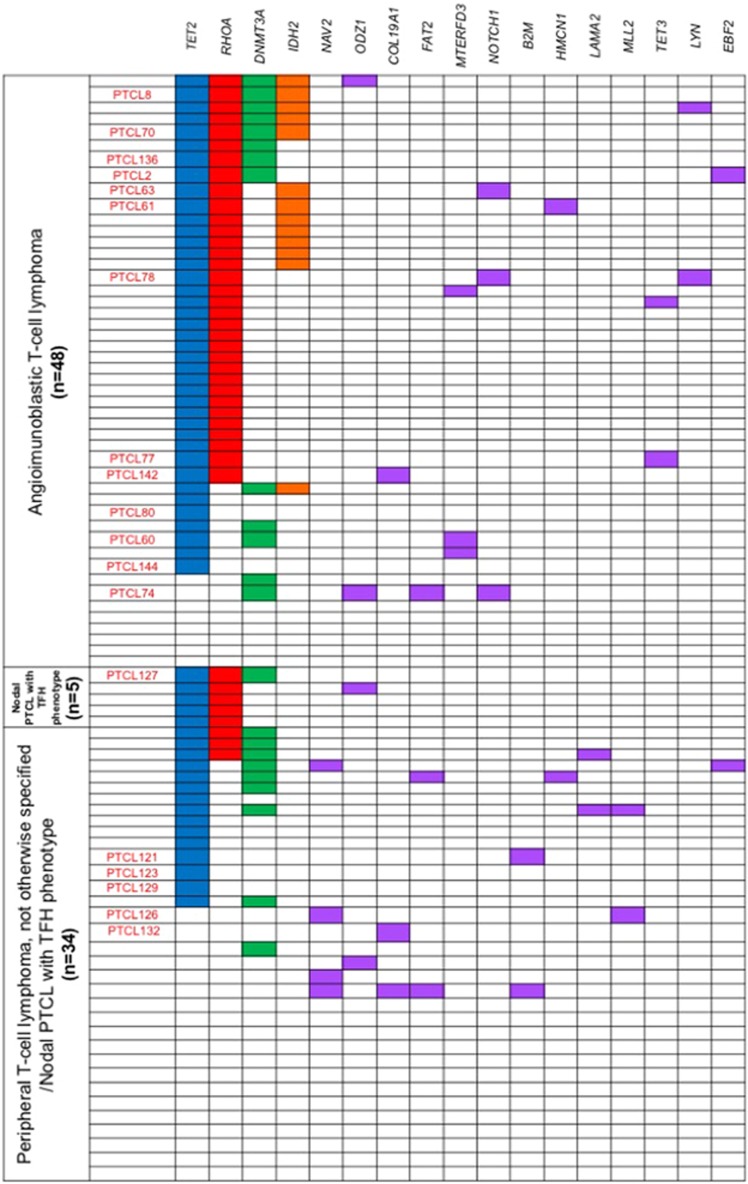
Targeted sequencing result of 87 nodal T-cell lymphoma samples. *TET2-*, *RHOA-*, *IDH2-* and *DNMT3A-*mutated cases are indicated by blue, red, orange and green boxes, respectively. Other recurrently mutated genes are in purple. The laser-microdissected samples are indicated in red letters.

Targeted sequencing for 71 genes was performed in 87 samples (Supplementary Table S1), including AITL (*n*=48), nodal PTCL with TFH phenotype (*n*=5) and PTCL-NOS/nodal PTCL with TFH phenotype (*n*=34). *TET2*, *DNMT3A*, *RHOA* and *IDH2* mutations were identified in 60 (68.7%), 23 (26.4%), 41 (47.1%) and 13 (14.9%) of 87 cases, respectively (Figure 1, Table 1, Supplementary Table S3). The mutational profiles of these 4 genes in 79 of the 87 samples are described elsewhere.^7^

Thirty-four novel recurrent mutations were identified in 13 of the 71 (18.3%) genes and in 24 of the 87 (26.4%) cases (Figure 1, Table 1 and Supplementary Table S4). Mutations in genes associated with lymphoid malignancies, for example, *Notch homolog 1, translocation-associated* (*NOTCH1*), *β2 microglobulin* (*B2M*) and *mixed-lineage leukemia 2* (*MLL2*) were identified in 3, 2 and 2 cases, respectively. Mutations in *FAT atypical cadherin 2* (*FAT2*), a gene associating with several cancers, and those in *TET3*, a member of the *TET* gene family, were identified in 3 and 2 cases, respectively.

Nineteen of the 87 samples were analyzed by the use of LMD (Figure 1). The frequencies of *TET2*, *DNMT3A*, *RHOA* and *IDH2* mutations in the laser-microdissected samples were similar to those found in the entire cohort (*TET2* mutations, 16/19 (84.1%); *DNMT3A* mutations, 7/19 (36.8%); *RHOA* mutations, 10/19 (52.6%) and *IDH2* mutations, 4/19 (21.1%)) (Table 2). A total of 26 *TET2* mutations were found in 16 cases, while 2 *TET2* mutations were found in 10 samples each (Supplementary Table S5). *NOTCH1* and *COL19A1* mutations were identified in 3 and 2 cases, respectively. Other gene mutations, including *NAV2*, *ODZ1*, *FAT2*, *MTERFD3*, *B2M*, *HMCN1*, *MLL2*, *TET3* and *LYN*, were identified in a single case each.

### Specific existence of G17V RHOA mutations in tumor cell-enriched cells of nodal T-cell lymphomas

**Figure 2:**
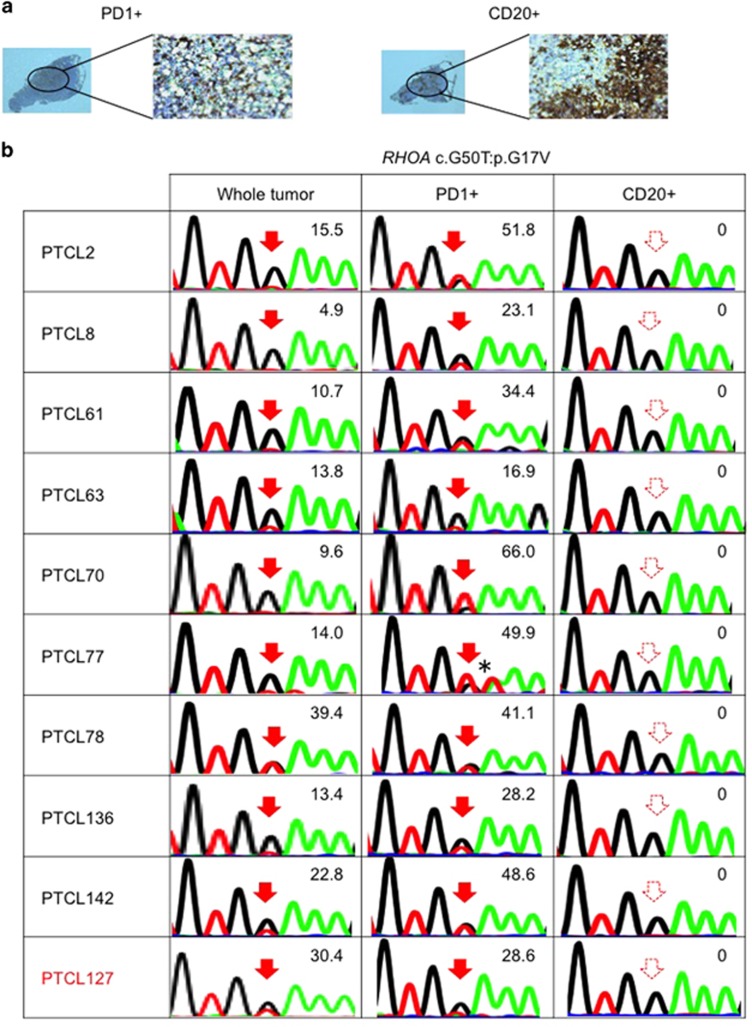
*RHOA* mutations are specific to PD1+ cells. (**a**) An example of the immunostaining pattern for PD1 and CD20 in AITL. Left, PD1+ cells; right, CD20+ cells. (**b**) Sequences of G17V *RHOA* mutations in whole tumor, PD1+ cells and CD20+ cells. The numeric values indicate allele frequencies of mutations defined by amplicon-based deep sequencing. The AITL samples are indicated in black letters. The nodal PTCL with TFH phenotype sample is indicated in red letters *: *RHOA* c.A51T:p.G17V, silent mutation. The filled and dashed red arrows indicate mutations and no mutations, respectively.

Previously, we reported that G17V *RHOA* mutations were detected by flow cytometry only in CD4-positive (CD4+) cells but not in other cell lineages purified from the skin tumor of a PTCL-NOS patient and the pleural effusion cells of an AITL patient.^8^ These preliminary results suggested that the G17V *RHOA* mutation may specifically exist in mature CD4+ T cells in PTCL-NOS and AITL. To gain further insight into the origin of the G17V *RHOA* mutation, we examined the mutation in laser-microdissected PD1+ and CD20+ B cells, which were assumed to be enriched and depleted in tumor cells, respectively, in 10 nodal T-cell lymphomas (1 nodal PTCL with TFH phenotype and 9 AITL cases). The G17V *RHOA* mutation was detected only in the PD1+ cells but not in the CD20+ cells in all 10 cases (Figure 2). The allele frequencies of the G17V *RHOA* mutations in the dissected PD1+ cells were substantially higher than those in the matched whole tumor samples in 7 of the 10 cases. The efficiency of mutation allele enrichment was not substantial in three cases (PTCL63, PTCL78 and PTCL127). In these cases, PD1+ cell selection was not sucessful enough to purify the tumor cells because of the presumed abundance of PD1+ non-tumor cells or the very high tumor cell content before the selection. Additionally, using flow cytometry, we found an AITL case showing that the G17V *RHOA* mutation existed in PD1+CD4+ cells sorted from bone marrow mononuclear cells (Supplementary Figure S2). This finding strongly supports our hypothesis that the acquisition of the G17V *RHOA* mutation is a specific event in TFH cells.

### Distribution of TET2, IDH2 and DNMT3A mutations

**Figure 3:**
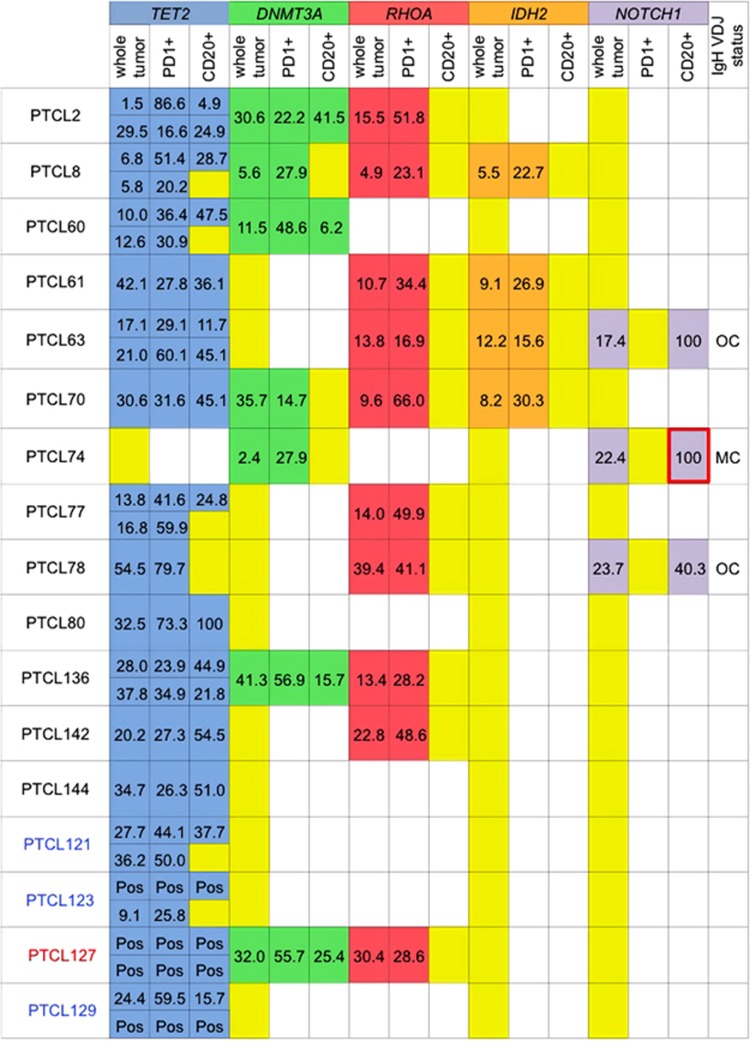
Distributions of *TET2/DNMT3A/RHOA/IDH2/NOTCH1* mutations and IgH VDJ status. Allele frequencies of *TET2/DNMT3A/RHOA/IDH2/NOTCH1* mutations in whole tumor, PD1+ cells and CD20+ cells are shown. The blue boxes represent positive *TET2* mutations; the green boxes, positive *DNMT3A* mutations; the red boxes, positive *RHOA* mutations; the orange boxes, positive *IDH2* mutations; the purple boxes, positive *NOTCH1* mutations; the yellow boxes, no mutations; and the white boxes, not examined. The numeric values indicate allele frequencies of mutations defined by deep sequencing, except for that in the box surrounded by bold red lines which was estimated by Sanger sequencing. IgH VDJ status indicates the IgH VDJ rearrangement status in whole-tumor-derived DNA. The AITL samples are indicated in black letters. The nodal PTCL with TFH phenotype sample is indicated in red letters. The PTCL-NOS/nodal PTCL with TFH phenotype sample is indicated in blue letters. MC, monoclonality; OC, oligoclonality; Pos: positivity was evaluated only by Sanger sequencing. Multiple *TET2* mutations were identified in PTLC2, 8, 60, 63, 77, 136, 121, 123, 127 and 129.

We and others have previously reported that *TET2* and *DNMT3A* mutations were found in apparently normal blood cells, including bone marrow mononuclear cells, and in immature progenitors and blood cells of various lineages isolated from peripheral blood of a few PTCL patients.^7, 9, 15, 16, 17^ We examined the distribution of *TET2*, *IDH2* and *DNMT3A* mutations in PD1+ and CD20+ cells. Twenty of the 26 *TET2* mutations were identified in both the PD1+ and the CD20+ cells (Supplementary Table S7), and 15 of the 16 *TET2*-mutated samples had at least one mutation in both the PD1+ and the CD20+ cells (Figure 3). Concomitantly, *DNMT3A* mutations were identified in both the PD1+ and CD20+ cells in four of the seven *DNMT3A*-mutated samples (Figure 3, Supplementary Table S8). In myeloid malignancies, *TET2* and *IDH2* mutations are known to be mutually exclusive.^14, 29^ However, we and others reported that *IDH2* mutations often coexist with *TET2* mutations in PTCL.^8, 10, 30^ *IDH2* mutations were identified in PD1+ cells but not in CD20+ cells in all 4 *TET2-* and *IDH2*-comutated samples (PTCL8, PTCL61, PTCL63 and PTCL70) (Figure 3). Each of these samples had at least one *TET2* mutation in both the PD1+ and CD20+ cells and the G17V *RHOA* mutation only in the PD1+ cells. That is, *TET2*, *IDH2* and G17V *RHOA* mutations coexisted in the PD1+ cells in these cases. In addition, we also found the coexistence of *IDH2*, *TET2* and G17V *RHOA* mutations in PD1+CD4+ cells sorted from the bone marrow mononuclear cells of an AITL patient (Supplementary Figure S2).

### B-cell-specific mutations in nodal T-cell lymphomas

**Figure 4:**
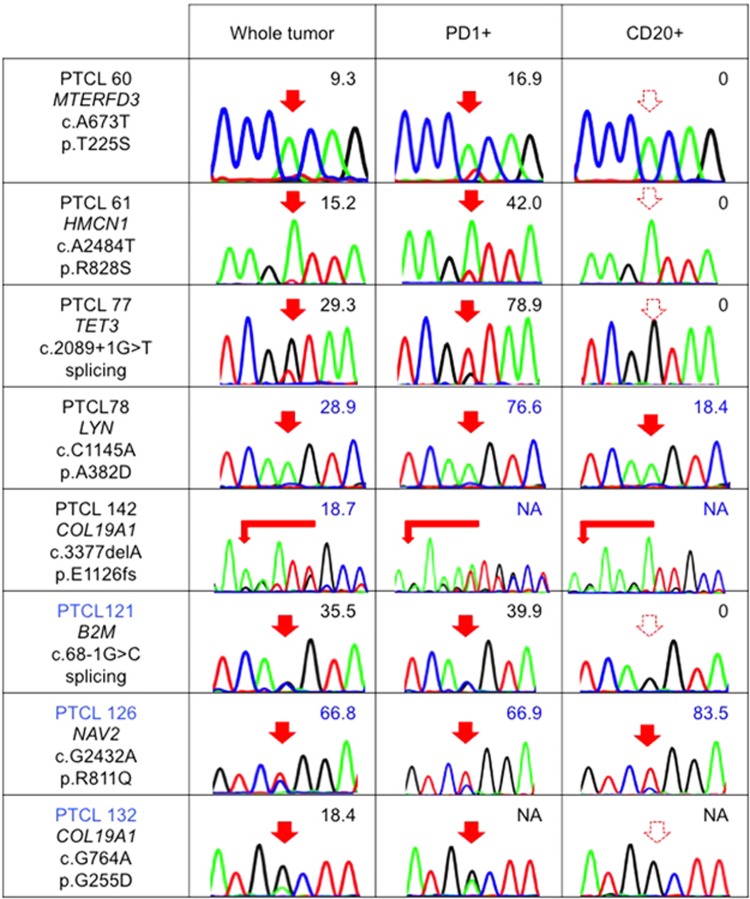
Distribution of newly identified gene mutations in nodal T-cell lymphomas. The results of Sanger sequencing and/or amplicon-based deep sequencing for some newly identified gene mutations in whole tumor, PD1+ cells and CD20+ cells are shown. The numeric values indicate allele frequencies of mutations defined by deep sequencing. The AITL samples are indicated in black letters. The PTCL-NOS/nodal PTCL with TFH phenotype sample is indicated in blue letters. NA, not analyzed by deep sequencing. The filled and dashed red arrows indicate mutations and no mutations, respectively.

To clarify the cellular origin of newly identified gene mutations, we also checked the distribution of these mutations in PD1+ and CD20+ cells (Table 2). We identified *B2M*, *COL19A1*, *HMCN1*, *MTERFD3* and *TET3* mutations only in PD1+ cells but not in CD20+ cells. *COL19A1*, *LYN*, *NAV2* and *NOTCH2NL* mutations were identified in both the PD1+ and CD20+ cells (Figure 4).

**Figure 5:**
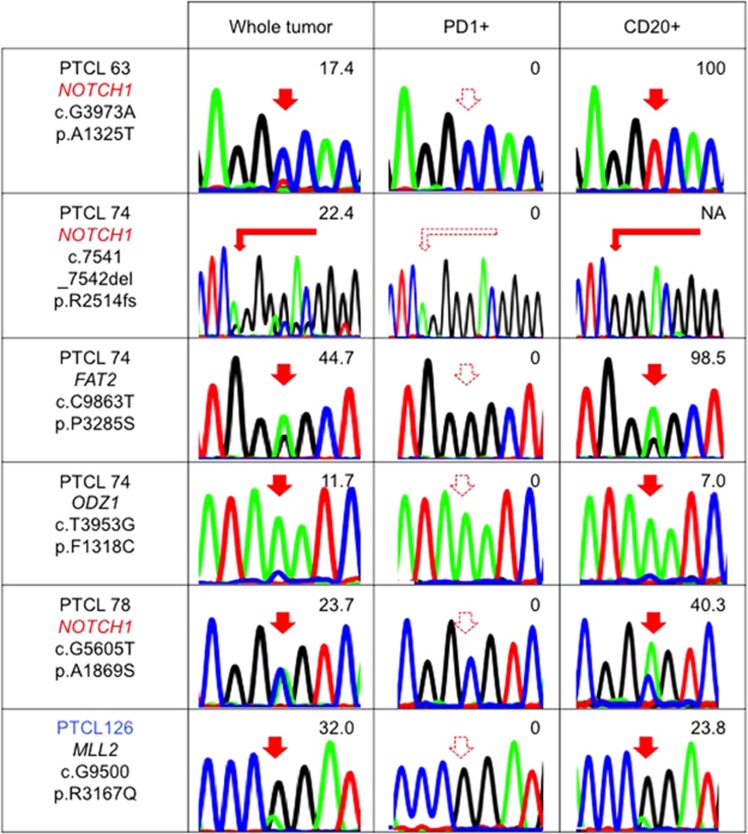
B-cell-specific mutations in nodal T-cell lymphomas. The results of Sanger sequencing and/or amplicon-based deep sequencing for some newly identified gene mutations in whole tumor, PD1+ cells and CD20+ cells are shown. The numeric values indicate allele frequencies of mutations defined by deep sequencing. The AITL samples are indicated in black letters. The PTCL-NOS/nodal PTCL with TFH phenotype sample is indicated in blue letters. NA, not analyzed by deep sequencing. The filled and dashed red arrows indicate mutations and no mutations, respectively. NOTCH1 is marked by red letters because this is repetitive.

Interestingly, three *NOTCH1* and one *FAT2*, *MLL2* and *ODZ1* mutations each were found only in the CD20+ but not in the PD1+ cells in four samples (PTCL 63, PTCL70, PTCL78 and PTCL128) (Figure 5). Especially, all three *NOTCH1* mutations identified by targeted sequencing were identified only in the CD20+ cells with high allele frequencies. The *NOTCH1* gene encodes a transmembrane protein. One of the *NOTCH1* mutations was a frameshift mutation residing in the PEST domain of the Notch1 protein. This would be an activating mutation, because deletion of the PEST domain enhances Notch signaling after ligand binding.^19^ The other two mutations were located in one of the epidermal growth factor-like and in the ankyrin repeat domains (Supplementary Figure S1). One of the *NOTCH1*-mutated samples simultaneously had two *TET2* mutations and G17V *RHOA* mutation (PTCL 63, Supplementary Table S9). In this case, both *TET2* mutations were detected in both the PD1+ and CD20+ cells, while the G17V *RHOA* mutation was confined to the PD1+ cells. We used the multiplex PCR method^26^ to also check the clonality of immunoglobulin genes in the samples with B-cell-specific mutations. Interestingly, only one sample showed monoclonal rearrangement while the others showed oligoclonal rearrangement (Table 3).

## Discussion

By determining the distribution of the mutations, we elucidated the clonal architecture of nodal T-cell lymphomas. *RHOA* mutations were identified only in PD1+ cells in 100% cases, while *TET2* and *DNMT3A* mutations were identified in both the PD1+ cells and CD20+, tumor-cell-depleted cells in the majority of cases. In addition, *IDH2* mutations were actually found only in the PD1+ cells and coexisted with *TET2* mutations. These data suggest that, in nodal T-cell lymphoma development, multistep tumorigenesis may progress in association with the differentiation of blood cells/lymphocytes. Surprisingly, some of the mutations resided in a B-cell-specific manner.

Recent genetic studies have revealed that, in several hematological cancers, several gene mutations existed in preleukemic hematopoietic cells as well as in tumor cells;^31^ examples are *TET2* and/or *DNMT3A* mutations in acute myeloid leukemia^32, 33, 34^ and *NOTCH1* and *SF3B1* mutations in chronic lymphocytic leukemia.^35, 36^ Moreover, somatic mutations have been demonstrated in elderly individuals without hematological malignancies: *DNMT3A*, *ASXL1*, and *TET2* mutations frequently observed in hematological malignancies were the most frequent in these cohorts.^37, 38, 39, 40^ Similarly, our data indicated that in nodal T-cell lymphomas, premalignant cells having *TET2* and/or *DNMT3A* mutations may differentiate not only into T-lineage tumor cells but also into B cells. In contrast, the G17V *RHOA* mutations specifically existed in the T cells of nodal T-cell lymphomas in all 13 cases (11 cases have been described in this paper, while 2 were previously described elsewhere^8^), indicating that the G17V *RHOA* mutation is the event after the B- and T-cell specification. This could happen right after the T/B specification, after differentiation into TFH cells or even after malignant transformation establishing a subclone. One possibility is that the G17V *RHOA* mutation occurs in *TET2*-mutated premalignant cells and facilitates the selective differentiation of *TET2*-mutated premalignant cells into tumor cells with the TFH phenotype. This needs to be proven in the future.

*IDH2* mutations were also specifically identified in the tumor-cell-enriched cells, suggesting that *IDH2* mutations are also tumor-cell-specific events in AITL, although the number of samples was not large enough to allow a definite conclusion. We have previously showed that the *IHD2*-mutated cases were almost a subcohort of G17V *RHOA*-mutated cases.^8^ This result could be interpreted that the acquisition of *IDH2* mutations may be the event occurring after the acquisition of *RHOA* mutation and thus the *IDH2*-mutated cells may, at least in some cases such as PTCL70, constitute a subclone in the RHOA-mutated clone. *TET2-* and *IDH2-*comutated AITL samples were reported to have more extensive histone modification profiles than those with *TET2* mutations without an *IDH2* mutation, while the difference in genome-wide cytosine methylation profiles between these samples was only moderate.^30^

Our data showed that B cells that have infiltrated AITL tissues also have gene mutations: the multilineal mutations represented by those in *TET2* and *DNMT3A*, and B-cell-specific mutations represented by those in *NOTCH1* and other genes. Monoclonal or oligoclonal expansion of B cells has been found in up to 30% of AITL cases.^41, 42, 43^ Furthermore, approximately 10% of AITL cases develop B-cell malignancies during their clinical course.^42, 44, 45, 46, 47^ Some lymphoma cells are infected by Epstein–Barr virus. In such cases, Epstein–Barr virus is proposed to contribute to the transformation of B cells.^45, 46^ This hypothesis, however, needs to be re-evaluated because Epstein–Barr virus was not detected in a substantial proportion of B-cell malignancies accompanying AITL.^48^ *TET2* mutations are found in diffuse large B-cell lymphomas.^49^ *Tet2*-deficient mice show the expansion of both B- and T-cell lineages in addition to prominent myeloproliferation.^15^ Combinational loss of *Tet1* and *Tet2* provokes B-cell malignancies in mice.^50^ Activating *NOTCH1* mutations were reported in diffuse large B-cell lymphomas,^51^ chronic lymphocytic leukemia,^52^ mantle cell lymphoma^22^ and follicular lymphoma.^53^ In our cohort, all three *NOTCH1* mutations were defined only in B cells with very high allele frequencies (Figure 5, Supplementary Table S9) and two of the three samples showed oligoclonality of B cells (Table 3). This implies that the origin of *NOTCH1* mutation is earlier than the acquisition of hypermutation of the CDR3 region in the immunoglobulin gene. Anyway, acquisition of these mutations in B-cell lineage may account for the frequent occurrence of B-cell lymphomas in AITL. Moreover, our data alert us to the need for careful interpretation of the mutational profiles of PTCLs because some of the mutations may not exist in tumor cells.

In conclusion, our findings illustrate the concept of multistep and multilineal tumorigenesis in nodal T-cell lymphomas (Supplementary Figure S3). Understanding the pathogenesis will lead us to better management of nodal T-cell lymphomas in future.

## Figures and Tables

**Table 1:** Targeted sequencing result of 87 nodal T-cell lymphoma samples

*Gene*	*AITL (*n=*48)*		*Nodal PTCL with TFH phenotype (*n=*5)*		*PTCL-NOS/nodal PTCL with TFH phenotype (*n=*34)*		*All (*n=*87)*	
	n	*%*	n	*%*	n	*%*	n	*%*
*TET2*	36	75	5	100	19	55.9	60	69
*RHOA*	33	68.8	5	100	3	8.8	41	47.1
*DNMT3A*	11	22.9	1	20	11	32.4	23	26.4
*IDH2*	13	27.1	0	0	0	0	13	14.9
*NAV2*	0	0	0	0	4	11.8	4	4.6
*ODZ1*	2	4.2	1	20	1	2.9	4	4.6
*COL19A1*	1	2.1	0	0	2	5.9	3	3.4
*FAT2*	1	2.1	0	0	2	5.9	3	3.4
*MTERFD3*	2	4.2	0	0	1	2.9	3	3.4
*NOTCH1*	3	6.3	0	0	0	0	3	3.4
*B2M*	0	0	0	0	2	5.9	2	2.3
*HMCN1*	1	2.1	0	0	1	2.9	2	2.3
*LAMA2*	0	0	0	0	2	5.9	2	2.3
*MLL2*	0	0	0	0	2	5.9	2	2.3
*TET3*	2	4.2	0	0	0	0	2	2.3
*LYN*	2	4.2	0	0	0	0	2	2.3
*EBF2*	1	2.1	0	0	1	2.9	2	2.3

Abbreviations: AITL, angioimmunoblastic T-cell lymphoma; nodal PTCL with TFH phenotype, nodal peripheral T-cell lymhoma with T follicular helper phenotype; PTCL-NOS, peripheral T-cell lymhoma, not otherwise specified.

**Table 2:** Mutation profiles of 19 laser microdissected samples

*Gene*	*AITL (*n=*13)*		*Nodal PTCL with TFH phenotype (*n=*1)*		*PTCL-NOS/nodal PTCL with TFH phenotype (*n=*5)*		*All (*n=*19)*	
	n	*%*	n	*%*	n	*%*	n	*%*
*TET2*	12	92.3	1	100	3	60	16	84.2
*RHOA*	9	69.2	1	100	0	0	10	52.6
*DNMT3A*	6	46.2	1	100	0	0	7	36.8
*IDH2*	4	30.8	0	0	0	0	4	21.1
*NAV2*	0	0	0	0	1	20	1	5.3
*ODZ1*	1	7.7	0	0	0	0	1	5.3
*COL19A1*	1	7.7	0	0	1	20	2	10.5
*FAT2*	1	7.7	0	0	0	0	1	5.3
*MTERFD3*	1	7.7	0	0	0	0	1	5.3
*NOTCH1*	3	23.1	0	0	0	0	3	15.8
*B2M*	0	0	0	0	1	20	1	5.3
*HMCN1*	1	7.7	0	0	0	0	1	5.3
*MLL2*	0	0	0	0	1	20	1	5.3
*TET3*	1	7.7	0	0	0	0	1	5.3
*LYN*	1	7.7	0	0	0	0	1	5.3

Abbreviations: AITL, angioimmunoblastic T-cell lymphoma; nodal PTCL with TFH phenotype, nodal peripheral T-cell lymhoma with T follicular helper phenotype; PTCL-NOS, peripheral T-cell lymhoma, not otherwise specified.

**Table 3:** VDJ rearrangement status of B-cell clones in B-cell-specific mutated samples

*Sample*	*Diagnosis*	*Number of colonies having the identical VDJ gene usage/total number of colonies analyzed*	*Common VDJ gene usage*	*Identity of V gene (%)*	*Amino-acid sequences of junctions*
PTCL63	AITL	2/12	V3-21/D2-2/J5	72.2	CARSTQTYYQLLWNG#NWFDPW^a^
PTCL74	AITL	NA^b^	V1-2/J1 or J2 or J3	84.4	Not identified at http://www.imgt.org
PTCL78	AITL	2/12	V3-23/J6/D4-17	72.2	CAKGNDYGDSYYYGMDVW
		2/12	V3/J6/D3-10	77.2	CARDRGYYYYGMDVW
PTCL126	PTCL-NOS/nodal PTCL with TFH phenotype	2/12	V6-1/J6/D3-3	71.0	CARTTPSTIFGVVTAGYYYYGMDVW

a Out of frame junction.

b NA, not applicable because direct sequencing demonstrated monoclonality.
