# Supplementary material for: Identification of cell-type-specific mutations in nodal T-cell lymphomas
Source: Blood Cancer J. 2017 Jan 6;7(1):e516–. doi: 10.1038/bcj.2016.122 (PMC5301031; doi:10.1038/bcj.2016.122)
Supplement: Supplementary Information [file bcj2016122x1.docx]

**Supplementary information**

**Identification of Cell-type-specific Mutations in Nodal T-cell lymphomas**

**Tran B. Nguyen, Mamiko Sakata-Yanagimoto, Yukitsugu Asabe, Daisuke Matsubara, Junko Kano, Kenichi Yoshida, Yuichi Shiraishi, Kenichi Chiba, Hiroko Tanaka, Satoru Miyano, Koji Izutsu, Naoya Nakamura, Kengo Takeuchi, Hiroaki Miyoshi, Koichi Ohshima, Takashi Minowa, Seishi Ogawa, Masayuki Noguchi and Shigeru Chiba**

**Contents:**

- Supplementary methods
- Supplementary figures and legends
- Supplementary tables
- Supplementary references

**Supplementary methods**

**Sorting of tumor cell-enriched fraction and other fractions**

Mononuclear cells (MNCs) were isolated from peripheral blood (PB) of the patient using Ficoll-Paque^TM^ PLUS (GE healthcare) following the manufacture’s protocol. MNCs were stained by fluorescein isothiocyanate (FITC)-conjugated anti-CD4 (BD Biosciences, 555346), anti-CD14 antibody (BD Biosciences, 555397), and phycoerythrin (PE)-conjugated anti-CD8 (Dako, clone DK25), anti-CD19 antibody (Dako, clone HD37), and allphycocyanin (APC)-conjugated anti-CD279/PD1 antibody (Bio Legend, clone EH12.2H7), and then fractionated by FACS Aria (BD Biosciences).

# Genomic DNA of the sorted cells was directly amplified using Repli G single cell kit (Qiagen). The DNA solution was diluted 100 times and 2ul of this was used for PCR under the following conditions: 94^o^C for 2 minutes, 35 cycles of 98^o^C for 10 seconds and 68^o^C for 30 seconds by KOD -Plus- Neo kit (Toyobo) with each primer set (Supplementary Table S6). PCR amplicons were used for amplicon-based sequencing and Sanger sequencing.

**Supplementary figures and legends**

**
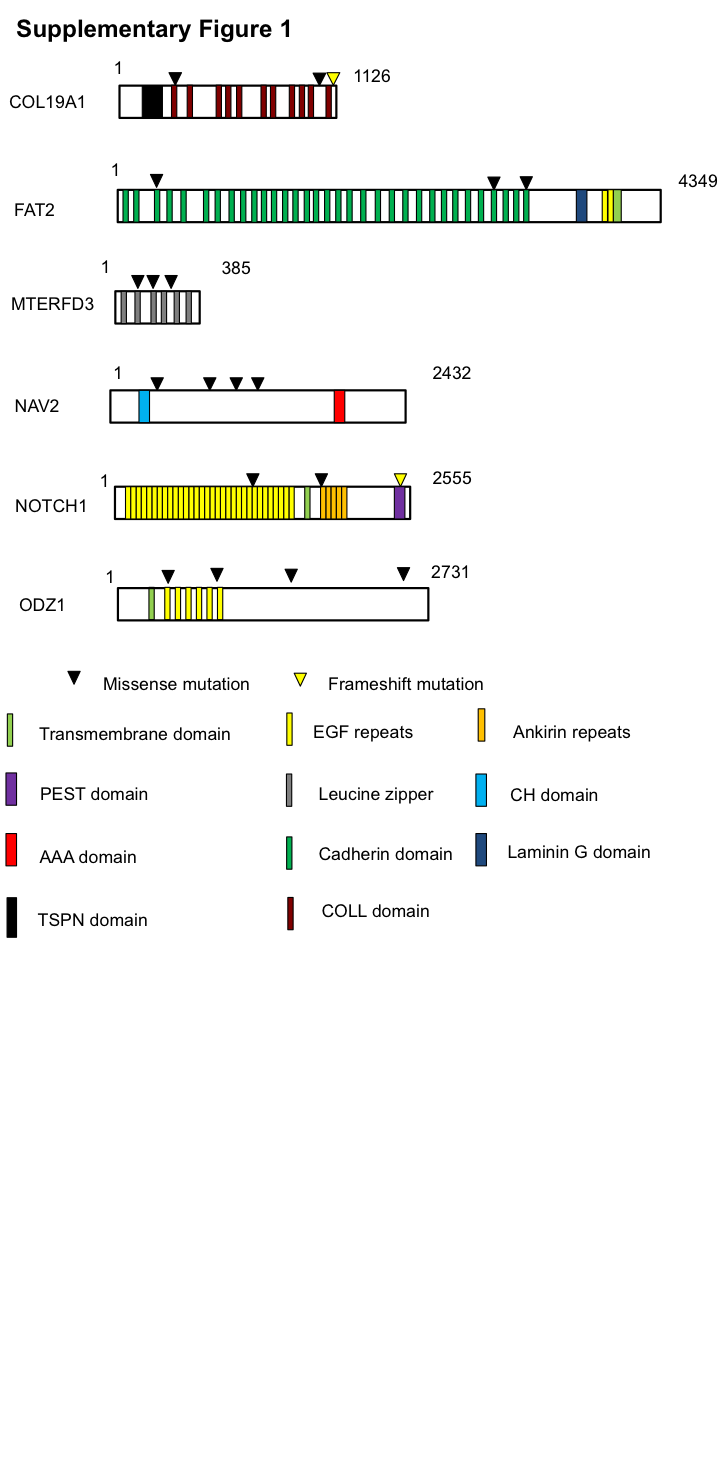
**

**Supplementary Figure 1:** Positions of mutations in COL19A1, FAT2, MTERFD3, NAV2, Notch1, and ODZ1 proteins.

**
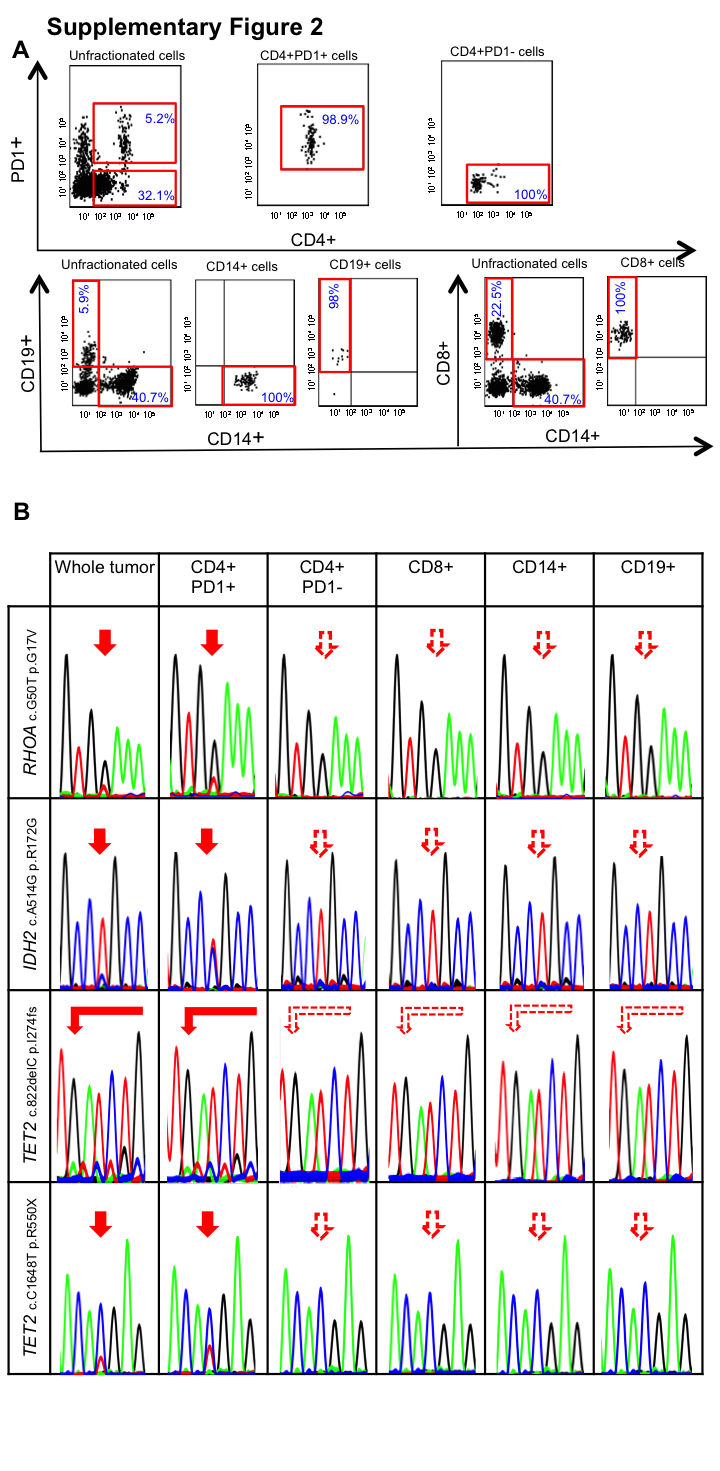
**

**Supplementary Figure 2:** Co-existence of *TET2* mutations with *IDH2* and *RHOA* mutations in a case of angioimmunoblastic T-cell lymphoma analyzed by flowcytometry

1. Frequencies of CD4+PD1+, CD4+PD1-, CD8+, CD14+, and CD19+ cells in bone marrow of one AITL sample analyzed by flowcytometry.
2. Sanger sequences of mutations in whole tumor, CD4+PD1+, CD4+PD1-, CD8+, CD14+, and CD19+ cells.

**
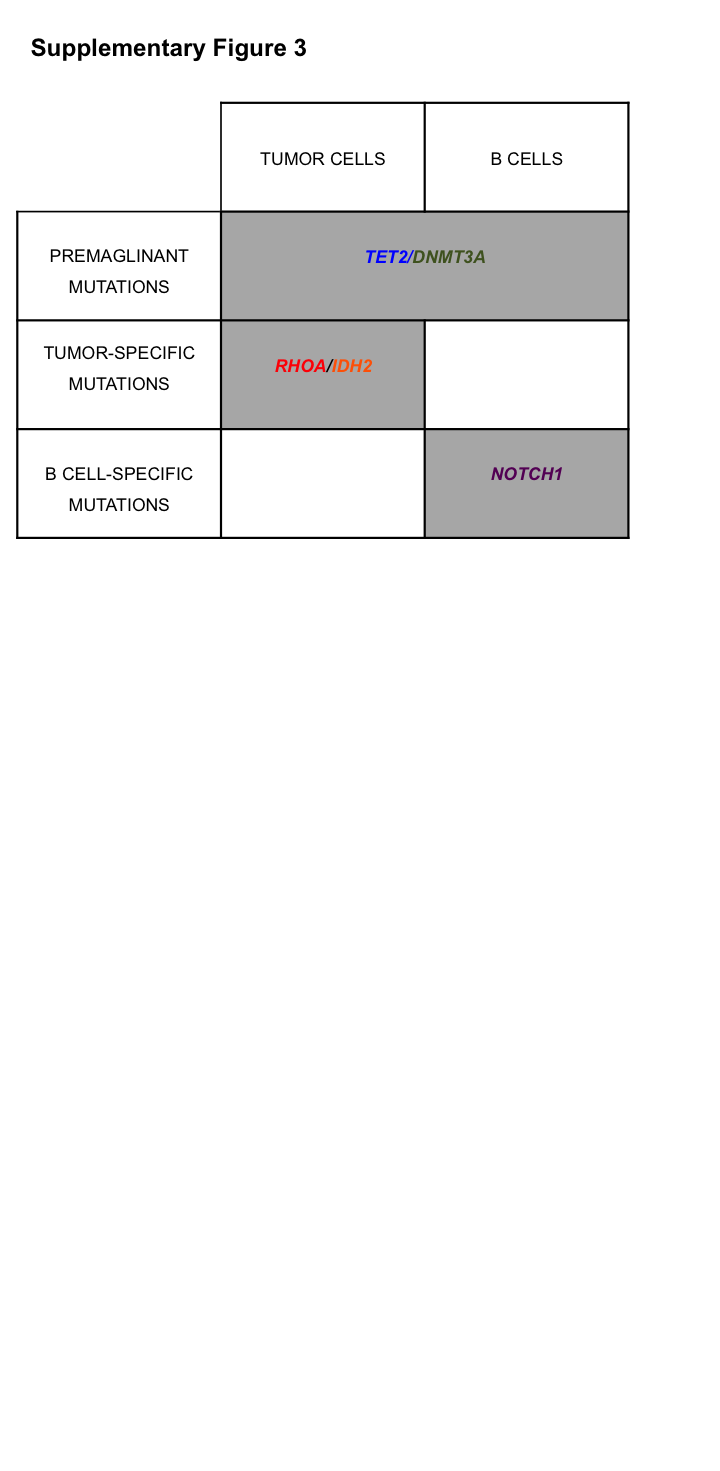
**

**Supplementary Figure 3:** Multi-step and multi-lineage of tumorigenesis in angioimmunoblastic T cell lymphomas. *TET2/DNMT3A* mutations were identified in both tumor and B cells as premalignant mutations while *RHOA/IDH2* mutations were confined in tumor cells as tumor-specific mutations. Moreover, *NOTCH1* mutations were identified only in B cells as B cell-specific mutations.

**Supplementary Table 1: Lists of Samples**

| **Number** | **Sample** | **Status of DNA** | **Diagnosis** |
| --- | --- | --- | --- |
| PTCL1 | Tumor | REPLI-g* | AITL |
| PTCL2 | Tumor | REPLI-g | AITL |
| PTCL3 | Tumor | REPLI-g | PTCL-NOS/ Nodal PTCL with TFH phenotype |
| PTCL4 | Tumor | REPLI-g | AITL |
| PTCL5 | Tumor | REPLI-g | PTCL-NOS/ Nodal PTCL with TFH phenotype |
| PTCL6 | Tumor | REPLI-g | Nodal PTCL with TFH phenotype |
| PTCL7 | Tumor | REPLI-g | AITL |
| PTCL8 | Tumor | REPLI-g | AITL |
| PTCL10 | Tumor | REPLI-g | PTCL-NOS/ Nodal PTCL with TFH phenotype |
| PTCL11 | Tumor | REPLI-g | PTCL-NOS/ Nodal PTCL with TFH phenotype |
| PTCL12 | Tumor | REPLI-g | PTCL-NOS/ Nodal PTCL with TFH phenotype |
| PTCL13 | Tumor | REPLI-g | AITL |
| PTCL14 | Tumor | REPLI-g | PTCL-NOS/ Nodal PTCL with TFH phenotype |
| PTCL15 | Tumor | original | PTCL-NOS/ Nodal PTCL with TFH phenotype |
| PTCL16 | Tumor | original | PTCL-NOS/ Nodal PTCL with TFH phenotype |
| PTCL17 | Tumor | original | PTCL-NOS/ Nodal PTCL with TFH phenotype |
| PTCL18 | Tumor | original | PTCL-NOS/ Nodal PTCL with TFH phenotype |
| PTCL19 | Tumor | original | PTCL-NOS/ Nodal PTCL with TFH phenotype |
| PTCL20 | Tumor | original | PTCL-NOS/ Nodal PTCL with TFH phenotype |
| PTCL21 | Tumor | original | PTCL-NOS/ Nodal PTCL with TFH phenotype |
| PTCL22 | Tumor | original | PTCL-NOS/ Nodal PTCL with TFH phenotype |
| PTCL23 | Tumor | original | PTCL-NOS/ Nodal PTCL with TFH phenotype |
| PTCL24 | Tumor | original | PTCL-NOS/ Nodal PTCL with TFH phenotype |
| PTCL25 | Tumor | original | AITL |
| PTCL26 | Tumor | original | AITL |
| PTCL27 | Tumor | original | AITL |
| PTCL28 | Tumor | original | AITL |
| PTCL30 | Tumor | original | AITL |
| PTCL31 | Tumor | original | AITL |
| PTCL33 | Tumor | original | AITL |
| PTCL34 | Tumor | original | AITL |
| PTCL35 | Tumor | original | AITL |
| PTCL36 | Tumor | original | AITL |
| PTCL37 | Tumor | original | AITL |
| PTCL38 | Tumor | original | AITL |
| PTCL39 | Tumor | REPLI-g | PTCL-NOS/ Nodal PTCL with TFH phenotype |
| PTCL40 | Tumor | REPLI-g | PTCL-NOS/ Nodal PTCL with TFH phenotype |
| PTCL41 | Tumor | REPLI-g | AITL |
| PTCL42 | Tumor | REPLI-g | AITL |
| PTCL43 | Tumor | REPLI-g | PTCL-NOS/ Nodal PTCL with TFH phenotype |
| PTCL44 | Tumor | REPLI-g | PTCL-NOS/ Nodal PTCL with TFH phenotype |
| PTCL45 | Tumor | REPLI-g | PTCL-NOS/ Nodal PTCL with TFH phenotype |
| PTCL46 | Tumor | REPLI-g | AITL |
| PTCL47 | Tumor | REPLI-g | PTCL-NOS/ Nodal PTCL with TFH phenotype |
| PTCL48 | Tumor | REPLI-g | AITL |
| PTCL49 | Tumor | REPLI-g | PTCL-NOS/ Nodal PTCL with TFH phenotype |
| PTCL50 | Tumor | REPLI-g | Nodal PTCL with TFH phenotype |
| PTCL51 | Tumor | REPLI-g | PTCL-NOS/ Nodal PTCL with TFH phenotype |
| PTCL52 | Tumor | REPLI-g | PTCL-NOS/ Nodal PTCL with TFH phenotype |
| PTCL53 | Tumor | REPLI-g | PTCL-NOS/ Nodal PTCL with TFH phenotype |
| PTCL54 | Tumor | REPLI-g | AITL |
| PTCL55 | Tumor | REPLI-g | AITL |
| PTCL56 | Tumor | REPLI-g | AITL |
| PTCL57 | Tumor | REPLI-g | AITL |
| PTCL58 | Tumor | REPLI-g | PTCL-NOS/ Nodal PTCL with TFH phenotype |
| PTCL59 | Tumor | original | AITL |
| PTCL60 | Tumor | original | AITL |
| PTCL61 | Tumor | original | AITL |
| PTCL62 | Tumor | original | AITL |
| PTCL63 | Tumor | original | AITL |
| PTCL64 | Tumor | original | AITL |
| PTCL65 | Tumor | original | AITL |
| PTCL66 | Tumor | original | AITL |
| PTCL67 | Tumor | original | AITL |
| PTCL68 | Tumor | original | AITL |
| PTCL69 | Tumor | original | Nodal PTCL with TFH phenotype |
| PTCL70 | Tumor | original | AITL |
| PTCL71 | Tumor | original | Nodal PTCL with TFH phenotype |
| PTCL72 | Tumor | original | AITL |
| PTCL73 | Tumor | original | AITL |
| PTCL74 | Tumor | original | AITL |
| PTCL75 | Tumor | original | AITL |
| PTCL76 | Tumor | original | PTCL-NOS/NODAL PTCL WITH TFH PHENOTYPE |
| PTCL77 | Tumor | original | AITL |
| PTCL78 | Tumor | original | AITL |
| PTCL79 | Tumor | original | AITL |
| PTCL80 | Tumor | original | AITL |
| PTCL121 | Tumor | original | PTCL-NOS/ Nodal PTCL with TFH phenotype |
| PTCL123 | Tumor | original | PTCL-NOS/ Nodal PTCL with TFH phenotype |
| PTCL126 | Tumor | original | PTCL-NOS/ Nodal PTCL with TFH phenotype |
| PTCL127 | Tumor | original | Nodal PTCL with TFH phenotype |
| PTCL129 | Tumor | original | PTCL-NOS/ Nodal PTCL with TFH phenotype |
| PTCL132 | Tumor | original | PTCL-NOS/ Nodal PTCL with TFH phenotype |
| PTCL136 | Tumor | original | AITL |
| PTCL142 | Tumor | original | AITL |
| PTCL144 | Tumor | original | AITL |
| PTCL159 | Tumor | original | PTCL-NOS/ Nodal PTCL with TFH phenotype |
| PTCL163 | Tumor | original | AITL |

***:** Whole genomic DNA was amplified by Repli-G kit (Qiagen), AITL: Angioimmunoblastic T-cell lymphoma, Nodal PTCL with TFH phenotype: nodal peripheral T-cell lymphoma with T follicular helper phenotype, PTCL-NOS: peripheral T-cell lymphoma, not otherwise specified.

**Supplementary Table 2: Genes Analyzed by Targeted Sequencing**

| **Genes analyzed by targeted sequencing** | | | |
| --- | --- | --- | --- |
| ACTB | EPHA6 | MLL2^*2^ | RHOC^*1^ |
| ACVR1C | FAT2 | MTERFD3 | RUNX1 |
| ADAMTS14 | GIMAP4 | MYO3A | SPTA1 |
| ADAMTS5 | GPI | NAV2 | SRGAP3 |
| ALDH1A2 | GRIK4 | NHS | ST18 |
| ANKRD5 | GRIP2 | NKAPL | STAB1 |
| B2M | HCLS1 | NOTCH1^*2^ | TCF20 |
| C9 | HMCN1 | NOTCH2^*2^ | TET1^*1^ |
| CACNA1D | HOXA2 | NOTCH3^*2^ | TET3^*1^ |
| CACNA1S | IQCJ-SCHIP1,SCHIP1 | ODZ1 | TLL1 |
| CASP3 | ITPR2 | PBX1 | TNFRSF14 |
| CCR10 | JAK3^*1^ | PEG3 |  |
| CD40LG | KCNMA1 | PKD2L1 |  |
| CDC42^*1^ | KIF21B | POLE |  |
| CDH10 | LAMA2 | PTEN |  |
| CLTC | LRP4 | PTPN23 |  |
| CNTN6 | LRRN3 | RAB9B |  |
| COL19A1 | LYN | RAC1^*1^ |  |
| CTTNBP2 | MAP2K1 | RAD21 |  |
| EBF2 | MAP3K3 | RHOB^*1^ |  |

Genes selected by whole-exome sequencing (unmarked), the family genes of those screened by whole-exome sequencing (^*1^), and genes susceptible to be mutated from the mutation profiles in other lymphoid malignancies (^*2^) were included.

**Supplementary Table 3: *TET2/DNMT3A/RHOA/IDH2* mutations of additional eight nodal T-cell lymphoma cases (not mentioned in reference paper^1^) identified by targeted sequencing**

| **Sample ID** | **Genes** | **Annotated genes** | **Mutation Type** | **Nucleotide Change** | **Amino acid Transcript** | **VAF*** |  |
| --- | --- | --- | --- | --- | --- | --- | --- |
| PTCL121 | TET2 | NM_001127208 | Nonsense | c.C2626T | p.Q876X | 0.2890 |  |
| PTCL121 | TET2 | NM_001127208 | Missense | c.G3866T | p.C1289F | 0.3080 |  |
| PTCL123 | TET2 | NM_001127208 | Frameshift | c.4657_4660del | p.1553_1554del | 0.0467 |  |
| PTCL123 | TET2 | NM_001127208 | Missense | c.A5642C | p.H1881P | 0.1000 |  |
| PTCL127 | DNMT3A | NM_153759 | Missense | c.G2078A | p.R693H | 0.3520 |  |
| PTCL127 | RHOA | NM_001664 | Missense | c.G50T | p.G17V | 0.2484 |  |
| PTCL127 | TET2 | NM_001127208 | Nonsense | c.C4889A | p.S1630X | 0.4340 |  |
| PTCL127 | TET2 | NM_001127208 | Nonsense | c.C346T | p.Q116X | 0.2340 |  |
| PTCL129 | TET2 | NM_001127208 | Frameshift | c.2188_2189del | p.730_730del | 0.2270 |  |
| PTCL129 | TET2 | NM_001127208 | Missense | c.T3965C | p.L1322P | 0.2300 |  |
| PTCL136 | DNMT3A | NM_153759 | Missense | c.G1175C | p.W392S | 0.3860 |  |
| PTCL136 | RHOA | NM_001664 | Missense | c.G50T | p.G17V | 0.0892 |  |
| PTCL136 | TET2 | NM_001127208 | Frameshift | c.1939_1940del | p.647_647del | 0.2130 |  |
| PTCL136 | TET2 | NM_001127208 | Nonsense | c.C2305T | p.Q769X | 0.0580 |  |
| PTCL142 | TET2 | NM_001127208 | Frameshift | c.3534_3540del | p.1178_1180del | 0.2020 |  |
| PTCL142 | RHOA | NM_001664 | Missense | c.G50T | p.G17V | 0.2107 |  |
| PTCL144 | TET2 | NM_001127208 | Missense | c.C4104G | p.F1368L | 0.3340 |  |
| PTCL144 | TET2 | NM_001127208 | Nonsense | c.C2305T | p.Q769X | 0.3700 |  |
| PTCL145 | TET2 | NM_001127208 | Nonsense | c.822delC | p.I274fs | 0.1780 |  |
| PTCL145 | TET2 | NM_001127208 | Nonsense | c.C1648T | p.R550X | 0.1740 |  |
| PTCL145 | RHOA | NM_001664 | Missense | c.G50T | p.G17V | 0.1300 |  |
| PTCL145 | IDH2 | NM_002168 | Missense | c.A514G | p.R172G | 0.2107 |  |
| ***** VAF: variant allele frequency | | | | | | |  |

**Supplementary Table 4: Other Gene Mutations than *TET2/DNMT3A/RHOA/IDH2* Identified by Targeted Sequencing**

| **Sample ID** | **Genes** | **Annotated genes** | **Mutation Type** | **Nucleotide Change** | **Amino acid Transcript** | **VAF** | **Exome^*1^** |
| --- | --- | --- | --- | --- | --- | --- | --- |
| PTCL03 | ACTA | NM_001100 | Nonsense | c.C1084T | p.Q362X | 0.1017 |  |
| PTCL02 | ACVR1C | NM_001111032 | Missense | c.C524A | p.A175D | 0.0909 | y |
| PTCL03 | ADAMTS14 | NM_080722 | Missense | c.T1816G | p.C606G | 0.0377 |  |
| PTCL05 | ADAMTS5 | NM_007038 | Missense | c.G1990A | p.V664M | 0.0750 | y |
| PTCL03 | ALDH1A2 | NM_170697 | Missense | c.A1143C | p.L381F | 0.0996 | y |
| PTCL05 | B2M | NM_004048 | Missense | c.A1G | p.M1V | 0.0831 | y |
| PTCL121 | B2M | NM_004048 | Splicing | c.C68-1G>C |  | 0.2900 |  |
| PTCL14 | CACNA1D | NM_000720 | Missense | c.C992T | p.T331M | 0.0425 |  |
| PTCL03 | CACNA1S | NM_000069 | Missense | c.G3190A | p.V1064I | 0.0543 | y |
| PTCL06 | CASP3 | NM_032991 | Missense | c.A392G | p.N131S | 0.1647 | y |
| PTCL02 | CDH10 | NM_006727 | Missense | c.C835T | p.L279F | 0.1017 | y |
| PTCL06 | CLTC | NM_004859 | Nonsense | c.T644G | p.L215X | 0.2099 | y |
| PTCL05 | COL19A1 | NM_001858 | Missense | c.C3121A | p.L1041I | 0.0892 | y |
| PTCL132 | COL19A1 | NM_001858 | Missense | c.G764A | p.G255D | 0.1650 |  |
| PTCL142 | COL19A1 | NM_001858 | Frameshift | c.3377delA | p.E1126fs | 0.1460 |  |
| PTCL132 | EBF1 | NM_024007 | Missense | c.G152C | p.R51P | 0.1900 |  |
| PTCL02 | EBF2 | NM_022659 | Missense | c.G100C | p.G34R | 0.2121 | y |
| PTCL14 | EBF2 | NM_022659 | Missense | c.G1186A | p.A396T | 0.0342 |  |
| PTCL02 | EPHA6 | NM_001080448 | Missense | c.G475A | p.E159K | 0.0776 | y |
| PTCL03 | FAT2 | NM_001447 | Missense | c.T1151C | p.V384A | 0.0558 |  |
| PTCL05 | FAT2 | NM_001447 | Missense | c.C10861T | p.H3621Y | 0.0819 | y |
| PTCL74 | FAT2 | NM_001447 | Missense | c.C9863T | p.P3285S | 0.4850 |  |
| PTCL05 | GPI | NM_000175 | Missense | c.A1594C | p.S532R | 0.0709 | y |
| PTCL05 | GRIK4 | NM_014619 | Missense | c.C541A | p.Q181K | 0.0815 | y |
| PTCL80 | GRIP2 |  | Unknown |  |  | 0.0368 |  |
| PTCL12 | GTF2IRD1 | NM_001199207 | Missense | c.C2843A | p.A948D | 0.3529 |  |
| PTCL03 | HMCN1 | NM_031935 | Missense | c.G14633C | p.S4878T | 0.1795 | y |
| PTCL61 | HMCN1 | NM_031935 | Missense | c.A2484T | p.R828S | 0.0803 |  |
| PTCL02 | HOXA2 | NM_006735 | Missense | c.T710C | p.L237P | 0.1186 | y |
| PTCL05 | IQCJ-SCHIP1 | NM_001197107 | Missense | c.G103A | p.G35S | 0.0646 | y |
| PTCL159 | LAMA2 | NM_0011079823 | Missense | c.A7804G | p.M2602V | 0.0452 |  |
| PTCL43 | LAMA2 | NM_000426 | Missense | c.T2054G | p.L685R | 0.3045 |  |
| PTCL03 | LRRN3 | NM_018334 | Missense | c.C986A | p.A329E | 0.0645 |  |
| PTCL15 | LTBP1 | NM_000627 | Missense | c.G2794T | p.G932W | 0.2581 |  |
| PTCL04 | LYN | NM_001111097 | Missense | c.C1015T | p.R339W | 0.0466 | y |
| PTCL78 | LYN | NM_001111097 | Missense | c.C1145A | p.A382D | 0.3052 |  |
| PTCL43 | MLL2 | NM_003482 | Missense | c.A10109G | p.Q3370R | 0.3784 |  |
| PTCL126 | MLL2 | NM_003482 | Missense | c.G9500A | p.R3167Q | 0.3400 |  |
| PTCL01 | MTERFD3 | NM_001033050 | Missense | c.C830T | p.T277I | 0.0820 | y |
| PTCL35 | MTERFD3 | NM_001033050 | Missense | c.G400A | p.E134K | 0.0865 |  |
| PTCL60 | MTERFD3 | NM_001033050 | Missense | c.A673T | p.T225S | 0.0955 |  |
| PTCL02 | MYO3A | NM_017433 | Missense | c.C2839T | p.H947Y | 0.0702 | y |
| PTCL05 | NAV2 | NM_001111018 | Missense | c.C2266T | p.R756C | 0.1250 | y |
| PTCL14 | NAV2 | NM_001111018 | Missense | c.G2315T | p.S772I | 0.0602 |  |
| PTCL22 | NAV2 | NM_001111018 | Missense | c.G106A | p.A36T | 0.2323 |  |
| PTCL126 | NAV2 | NM_001111018 | Missense | c.G2432A | p.R811Q | 0.3570 |  |
| PTCL63 | NOTCH1 | NM_017617 | Missense | c.G3973A | p.A1325T | 0.3946 |  |
| PTCL74 | NOTCH1 | NM_017617 | Nonsense | c.7541_c.7542del | p.2514Rfs | 0.2042 |  |
| PTCL78 | NOTCH1 | NM_017617 | Missense | c.G5605T | p.A1869A | 0.1654 |  |
| PTCL136 | NOTCH2NL | NM_203458 | Missense | c.A285C | p.Q95H | 0.1170 |  |
| PTCL26 | NOTCH3 | NM_000435 | Missense | c.C5542T | p.R1848C | 0.0508 |  |
| PTCL06 | ODZ1 | NM_014253 | Missense | c.G7831C | p.V2611L | 0.1547 |  |
| PTCL18 | ODZ1 | NM_001163278 | Missense | c.G2200A | p.G734S | 0.0685 |  |
| PTCL66 | ODZ1 | NM-001163278 | Missense | c.C1148A | p.S383Y | 0.0571 |  |
| PTCL74 | ODZ1 | NM_014253 | Missense | c.T3953G | p.F1318C | 0.1154 |  |
| PTCL06 | PEG3 | NM_001146185 | Missense | c.G3964A | p.G1322R | 0.2353 | y |
| PTCL05 | PKD2L1 | NM_001253837 | Missense | c.C391A | p.Q131K | 0.1444 | y |
| PTCL02 | PTEN | NM_000314 | Nonsense | c.C388T | p.R130X | 0.0666 | y |
| PTCL02 | PTPN23 | NM_015466 | Missense | c.A647G | p.E216G | 0.2692 | y |
| PTCL02 | RAB9B | NM_016370 | Missense | c.C381A | p.D127E | 0.0972 | y |
| PTCL02 | RAD21 | NM_006265 | Missense | c.C59T | p.A20V | 0.2195 | y |
| PTCL35 | RUNX2 | NM_004348 | Missense | c.C191A | p.A64E | 0.0741 |  |
| PTCL05 | SRGAP3 | NM_001033117 | Missense | c.G2081T | p.S694I | 0.0846 | y |
| PTCL05 | ST18 | NM_014682 | Missense | c.G2315C | p.G772A | 0.1831 | y |
| PTCL05 | STAB1 | NM_015136 | Missense | c.C4906T | p.R1636C | 0.1360 | y |
| PTCL144 | TCF20 | NM_005650 | Missense | c.G4223A | p.R1408K | 0.6420 |  |
| PTCL26 | TET3 | NM_144993 | Nonsense | c.G3900A | p.W1300X | 0.1172 |  |
| PTCL77 | TET3 | NM_144993 | Splicing | c.2089+1G>T |  | 0.1621 |  |
| PTCL03 | TLL1 | NM_012464 | Missense | c.A1790G | p.Y597C | 0.1057 |  |

^*1^Gene mutations also identified in whole-exome sequencing^1^.

**Supplementary Table 5: Mutation Profiles of Samples Analyzed by Laser Microdissection**

| **Sample ID** | **Genes** | **Annotated genes** | **Mutation Type** | **Nucleotide Change** | **Amino acid Transcript** | **VAF** |  |  |
| --- | --- | --- | --- | --- | --- | --- | --- | --- |
| PTCL2 | DNMT3A | NM_175629 | Splice site | c.2409-1G>A | p.V690splice | 0.4300 |  |  |
| PTCL2 | RHOA | NM_001664 | Missense | c.G50T | p.G17V | 0.0765 |  |  |
| PTCL2 | TET2 | NM_001127208 | Nonsense | c.G2263T | p.E755X | 0.2585 |  |  |
| PTCL2 | TET2 | NM_001127208 | Frameshift | c.5348delA | p.Q1783fs | 0.3692 |  |  |
| PTCL8 | DNMT3A | NM_175629 | Missense | c.G2207A | p.R736H | 0.0295 |  |  |
| PTCL8 | IDH2 | NM_002168 | Missense | c.G515C | p.R172T | 0.0778 |  |  |
| PTCL8 | RHOA | NM_001664 | Missense | c.G50T | p.G17V | 0.0748 |  |  |
| PTCL8 | TET2 | NM_001127208 | Frameshift | c.1355delA | p.E452fs | 0.0757 |  |  |
| PTCL60 | DNMT3A | NM_175629 | Missense | c.T2251G | p.F751V | 0.1067 |  |  |
| PTCL60 | MTERFD3 | NM_001033050 | Missense | c.A673T | p.T225S | 0.0955 |  |  |
| PTCL60 | TET2 | NM_001127208 | Frameshift | c.2475delA | p.S825fs | 0.1368 |  |  |
| PTCL60 | TET2 | NM_001127208 | Missense | c.A4160G | p.N1387S | 0.1594 |  |  |
| PTCL61 | HMCN1 | NM_031935 | Missense | c.A2484T | p.R828S | 0.0803 |  | |
| PTCL61 | IDH2 | NM_002168 | Missense | c.G515T | p.R172M | 0.0415 |  | |
| PTCL61 | RHOA | NM_001664 | Missense | c.G50T | p.G17V | 0.0296 |  | |
| PTCL61 | TET2 | NM_001127208 | Frameshift | c.4192_4193insT | p.L1398fs | 0.3290 |  |  |
| PTCL63 | IDH2 | NM_002168 | Missense | c.G515A | p.R172K | 0.2235 |  |  |
| PTCL63 | NOTCH1 | NM_017617 | Missense | c.G3973A | p.A1325T | 0.3946 |  |  |
| PTCL63 | RHOA | NM_001664 | Missense | c.G50T | p.G17V | 0.1897 |  |  |
| PTCL63 | TET2 | NM_001127208 | Nonsense | c.C2746T | p.Q916X | 0.2235 |  |  |
| PTCL63 | TET2 | NM_001127208 | Missense | c.G4133T | p.C1378F | 0.2271 |  |  |
| PTCL70 | DNMT3A | NM_175629 | Missense | c.G2207A | p.R736H | 0.2165 |  |  |
| PTCL70 | IDH2 | NM_002168 | Missense | c.G516C | p.R172S | 0.0694 |  |  |
| PTCL70 | RHOA | NM_001664 | Missense | c.G50T | p.G17V | 0.0562 |  |  |
| PTCL70 | TET2 | NM_001127208 | Frameshift | c.5477delA | p.E1826fs | 0.2987 |  |  |
| PTCL74 | FAT2 | NM_001447 | Missense | c.C9863T | p.P3285S | 0.4850 |  |  |
| PTCL74 | DNMT3A | NM_175629 | Splice site | c.2478+1G>- | p.L713splice | 0.0574 |  |  |
| PTCL74 | NOTCH1 | NM_017617 | Nonsense | c.7541_c.7542del | p.R2514fs | 0.2042 |  |  |
| PTCL74 | ODZ1 | NM_014253 | Missense | c.T3953G | p.F1318C | 0.1154 |  |  |
| PTCL77 | RHOA | NM_001664 | Missense | c.G50T | p.G17V | 0.1054 |  |  |
| PTCL77 | TET2 | NM_001127208 | Frameshift | c.5269delC | p.H1757fs | 0.0856 |  |  |
| PTCL77 | TET2 | NM_001127208 | Missense | c.G5687C | p.R1896T | 0.0922 |  |  |
| PTCL77 | TET3 | NM_144993 | Splicing | c.2089+1G>T |  | 0.1621 |  |  |
| PTCL78 | NOTCH1 | NM_017617 | Missense | c.G5605T | p.A1869S | 0.1654 |  | |
| PTCL78 | LYN | NM_001111097 | Missense | c.C1145A | p.A382D | 0.3052 |  |  |
| PTCL78 | RHOA | NM_001664 | Missense | c.G50T | p.G17V | 0.4531 |  |  |
| PTCL78 | TET2 | NM_001127208 | Nonsense | c.C2578T | p.Q860X | 0.6306 |  |  |
| PTCL80 | TET2 | NM_001127208 | Nonsense | c.C1630T | p.R544X | 0.2861 |  |  |
| PTCL121 | B2M | NM_004048 | Splicing | c.C68-1G>C |  | 0.2900 |  |  |
| PTCL121 | TET2 | NM_001127208 | Nonsense | c.C2626T | p.Q876X | 0.2890 |  |  |
| PTCL121 | TET2 | NM_001127208 | Missense | c.G3866T | p.C1289F | 0.3080 |  |  |
| PTCL123 | TET2 | NM_001127208 | Frameshift | c.4657_4660del | p.1553_1554del | 0.0467 |  |  |
| PTCL123 | TET2 | NM_001127208 | Missense | c.A5642C | p.H1881P | 0.1000 |  |  |
| PTCL126 | MLL2 | NM_003482 | Missense | c.G9500A | p.R3167Q | 0.3400 |  |  |
| PTCL126 | NAV2 | NM_001111018 | Missense | c.G2432A | p.R811Q | 0.3570 |  |  |
| PTCL127 | DNMT3A | NM_153759 | Missense | c.G2078A | p.R693H | 0.3520 |  |  |
| PTCL127 | RHOA | NM_001664 | Missense | c.G50T | p.G17V | 0.2484 |  |  |
| PTCL127 | TET2 | NM_001127208 | Nonsense | c.C4889A | p.S1630X | 0.4340 |  |  |
| PTCL127 | TET2 | NM_001127208 | Nonsense | c.C346T | p.Q116X | 0.2340 |  |  |
| PTCL129 | TET2 | NM_001127208 | Frameshift | c.2188_2189del | p.730_730del | 0.2270 |  |  |
| PTCL129 | TET2 | NM_001127208 | Missense | c.T3965C | p.L1322P | 0.2300 |  |  |
| PTCL132 | COL19A1 | NM_001858 | Missense | c.G764A | p.G255D | 0.1650 |  |  |
| PTCL136 | DNMT3A | NM_153759 | Missense | c.G1175C | p.W392S | 0.3860 |  |  |
| PTCL136 | RHOA | NM_001664 | Missense | c.G50T | p.G17V | 0.0892 |  |  |
| PTCL136 | NOTCH2NL | NM_203458 | Missense | c.A285C | p.Q95H | 0.1170 |  |  |
| PTCL136 | TET2 | NM_001127208 | Frameshift | c.1939_1940del | p.647_647del | 0.2130 |  |  |
| PTCL136 | TET2 | NM_001127208 | Nonsense | c.C2305T | p.Q769X | 0.0580 |  |  |
| PTCL142 | COL19A1 | NM_001858 | Frameshift | c.3377delA | p.E1126fs | 0.1460 |  |  |
| PTCL142 | RHOA | NM_001664 | Missense | c.G50T | p.G17V | 0.2107 |  |  |
| PTCL142 | TET2 | NM_001127208 | Frameshift | c.3534_3540del | p.1178_1180del | 0.2020 |  |  |
| PTCL144 | TET2 | NM_001127208 | Missense | c.C4104G | p.F1368L | 0.3340 |  |  |
| PTCL144 | TET2 | NM_001127208 | Nonsense | c.C2305T | p.Q769X | 0.3700 |  |  |

**Supplemental Table 6: Primers for PCR-based Deep Sequencing**

| **Patient ID** | **Gene** | **Forward** | **Reverse** |
| --- | --- | --- | --- |
| PTCL03 | ACTA | ATTTGCGGTGGACGATGGAA | CTGTCTTGCAGATCATCGCC |
| PTCL72 | ACTB | AGGAAAGGACAAGAAGCCCT | ACCATGGATGATGATATCGCCG |
| PTCL03 | ADAMTS14 | AGCCAGCAAGGTCATCCATT | CTGTGCTTGGCATTCTGGTG |
| PTCL03 | ANKRD5 | TTTCATGTCCAGTCCACAGGC | AGAAAAATGTCTGCCTTAGCGT |
| PTCL121 | B2M | TGTTGGGAAGGTGGAAGCTC | CGGATGGATGAAACCCAGACA |
| PTCL14 | CACNA1D | CTGGCAAGGGTTCTCACCTT | AAAGTTGGTGATGCCTCCGT |
| PTCL132 | COL19A1 | TCAGGCGGAAAGTGCAAAAG | TGCACTGGCACTGGTCTTTA |
| PTCL142 | COL19A1 | GCTTCTCCCCTAGGGTCCTT | GTGTTCAATTCCCACCTGTGC |
| PTCL14 | EBF2 | GAGATGCTGACCCCAAGCTG | TGAGTTTTGCACTTTATGCTGGAT |
| PTCL03 | FAT2 | GATAGTGGGCTCTGTCGTGG | GGGCTTTCACCTACCACCTT |
| PTCL05 | FAT2 | GAGCTGGTAGAAGCCCATCC | GGACACGCTGACCTATAGCC |
| PTCL74 | FAT2 | AAGAGCAAGGTGGCAGATCC | TCATTGCTGCTGTCACCTCTT |
| PTCL80 | GRIP2 | CTCTGGAGTCCCACGACAGT | CATCCATCATGACGGGCACC |
| PTCL12 | GTF2IRD1 | TCAACAGCCTTTTCCCCTCC | CACATCCCAGAGTAGACGGC |
| PTCL61 | HMCN1 | GTAATGTCTTCCAGCACCTCCA | GTTGGCTGATGGAACTAACTGA |
| PTCL159 | LAMA2 | TCAGAGCAGACGAACCTGTG | AGTTCGCTCTACATGAACGGAA |
| PTCL43 | LAMA2 | GGCACACATTTTCCAGTCCG | AAAATTCACTCCTGGGGCGG |
| PTCL03 | LRRN3 | GCTGTGGATAACCTGCCAGA | ACGGATGACACAGTCACACC |
| PTCL15 | LTBP1 | AGGATGTGTTGAGTGCCTCTG | AACTCACCCACACACCCTTG |
| PTCL78 | LYN | ATGGGGTCACATGTTCATGACT | GGAGGATTCCAAAGGACCACA |
| PTCL08 | MLL2 | GCGAAGTGTGGGCTAGAGAC | CCCCTGACCCTTATTCTCGC |
| PTCL43 | MLL2 | GCTGAGGTTACCTGTATCTGGG | TGGTGTCCAATCAAGGGCAT |
| PTCL126 | MLL2 | CAAGGTCAAAGCCCCACTCT | CCACCCTTCTCCTTGCCAAT |
| PTCL35 | MTERFD3 | TGGTGAAACCTTGCTCCTGG | GGCTCTGAGGCCAACATGAA |
| PTCL60 | MTERFD3 | GCAGCTGTCAAAAGTCTGCT | ACCGCTGTTAACACCCAGAG |
| PTCL14 | NAV2 | GTCGCTATGTGTACTCCGCC | TGCCAAGCCCTCTTTCTCTC |
| PTCL22 | NAV2 | GCCCTGAAAGCTCACCAATG | AGGACGCCATCTGTCACATC |
| PTCL126 | NAV2 | TGTGGACGTCTCAGACAAGG | AGCGTTTCCCTCCATGTCAC |
| PTCL04 | NHS1 | CCTGCCGCCAGGATTTTAGT | CCTACACCCTGGCCATTCTC |
| PTCL63 | NOTCH1 | CATCAGGGTGAGGAGGAGGATGA | GAGTCCGTCATCAATGGCTGC |
| PTCL74 | NOTCH1 | CCAGTCGGAGACGTTGGAAT | TGCACACTATTCTGCCCCAG |
| PTCL78 | NOTCH1 | AGTATCAACTGTACCCCAGCC | CTGGACGACCAGACAGACCA |
| PTCL136 | NOTCH2NL | CTCCCAGAATGGTGGGACTT | TGGGGGACATTTAAGAGCCAG |
| PTCL26 | NOTCH3 | TGAGGTCCAAAGTGTGTGCC | AGCTAGCATCATCTCCGACC |
| PTCL06 | ODZ1 | TCGACAGTTGTCCCATACCG | GGGTCTCTGGAGGAAGACCT |
| PTCL18 | ODZ1 | TGTAACCAACCCTCCTTCTCC | CTGGGTAGGACCAACATGTGA |
| PTCL66 | ODZ1 | TCTGTCCACGTAGAGGACTTC | TTAGCAAAGGGAACAGGGGG |
| PTCL74 | ODZ1 | CCAGTGATGCCTACCTGAGTG | TTGCTCTTTCCCAACAGGCA |
| PTCL26 | TET3 | CTTTGCCCAGAGCTCCAACT | CACCCACACCGTTAGTCCTC |
| PTCL77 | TET3 | CTGAGAACCCACTCACACCC | CCTTTCTCTGCCCTTCCCTG |
| PTCL03 | TLL1 | GAGTGTGCCAAACCTGACCG | GTGCAGTCATACCTTCACAGC |
| PTCL19 | ZSCAN18 | CCAATGCCTCGTCTGGGATT | AAGGAGAAAAGCTACGCGCT |

**Supplemental Table 7: *TET2* mutations in dissected samples were defined by Sanger sequencing and amplicon-based sequencing**

| **Sample** | **Mutation type** | **REFSEQ** | **Nucleotide change** | **Amino acid Transcript** | **Whole tumor** | | **PD1+** | | **CD20+** | |
| --- | --- | --- | --- | --- | --- | --- | --- | --- | --- | --- |
|  |  |  |  |  | **Sanger** | **(%)** | **Sanger** | **(%)** | **Sanger** | **(%)** |
| PTCL2 | Nonsense | NM_001127208 | c.G2263T | p.E755X | positive | 1.5 | positive | 86.6 | positive | 4.9 |
| PTCL2 | Frameshift | NM_001127208 | c.5348delA | p.Q1783fs | positive | 29.5 | positive | 16.6 | positive | 24.9 |
| PTCL8 | Frameshift | NM_001127208 | c.3501_3501del | p.1167_1167 del | positive | 6.8 | positive | 51.4 | positive | 28.7 |
| PTCL8 | Frameshift | NM_001127208 | c.1355delA | p.E452fs | positive | 5.8 | positive | 20.2 | negative | 0 |
| PTCL60 | Frameshift | NM_001127208 | c.2475delA | p.S825fs | positive | 10 | positive | 36.4 | positive | 47.5 |
| PTCL60 | Missense | NM_001127208 | c.A4160G | p.N1387S | positive | 12.6 | positive | 30.9 | negative | 0 |
| PTCL61 | Frameshift | NM_001127208 | c.4192_4193insT | p.L1398fs | positive | 42.1 | positive | 27.8 | positive | 36.1 |
| PTCL63 | Nonsense | NM_001127208 | c.C2746T | p.Q916X | positive | 17.1 | positive | 29.1 | positive | 11.7 |
| PTCL63 | Missense | NM_001127208 | c.G4133T | p.C1378F | positive | 21 | positive | 60.1 | positive | 45.1 |
| PTCL70 | Frameshift | NM_001127208 | c.5477delA | p.E1826fs | positive | 30.6 | positive | 31.6 | positive | 4.4 |
| PTCL77 | Frameshift | NM_001127208 | c.5269delC | p.H1757fs | positive | 13.8 | positive | 41.6 | positive | 24.8 |
| PTCL77 | Missense | NM_001127208 | c.G5687C | p.R1896T | positive | 16.8 | positive | 59.9 | negative | 0 |
| PTCL78 | Nonsense | NM_001127208 | c.C2578T | p.Q860X | positive | 54.5 | positive | 79.7 | negative | 0 |
| PTCL80 | Nonsense | NM_001127208 | c.C1630T | p.R544X | positive | 32.5 | positive | 73.3 | positive | 100 |
| PTCL121 | Nonsense | NM_001127208 | c.C2626T | p.Q876X | positive | 27.7 | positive | 44.1 | positive | 37.7 |
| PTCL121 | Missense | NM_001127208 | c.G3866T | p.C1289F | positive | 36.2 | positive | 50 | negative | 0 |
| PTCL123 | Frameshift | NM_001127208 | c.4657_4660del | p.1553_1554del | positive | NA | positive | ND | positive | NA |
| PTCL123 | Missense | NM_001127208 | c.A5642C | p.H1881P | positive | 9.1 | positive | 25.8 | negative | 0 |
| PTCL127 | Nonsense | NM_001127208 | c.C4889A | p.S1630X | positive | NA | positive | ND | positive | NA |
| PTCL127 | Nonsense | NM_001127208 | c.C346T | p.Q116X | positive | NA | positive | ND | positive | NA |
| PTCL129 | Frameshift | NM_001127208 | c.2188_2189del | p.730_730del | positive | 24.4 | positive | 59.5 | positive | 15.7 |
| PTCL129 | Missense | NM_001127208 | c.T3965C | p.L1322P | positive | NA | positive | ND | positive | NA |
| PTCL136 | Frameshift | NM_001127208 | c.1939_1940del | p.647_647del | positive | 28 | positive | 23.9 | positive | 44.9 |
| PTCL136 | Nonsense | NM_001127208 | c.C2305T | p.Q769X | positive | 37.8 | positive | 34.9 | positive | 21.8 |
| PTCL142 | Frameshift | NM_001127208 | c.3534_3540del | p.1178_1180del | positive | 20.2 | positive | 27.3 | positive | 54.5 |
| PTCL144 | Missense | NM_001127208 | c.C4104G | p.F1368L | positive | 34.7 | positive | 26.3 | positive | 51 |

***** NA: not analyzed.

**Supplemental Table 8**: ***DNMT3A* mutations in dissected samples were defined by Sanger sequencing and amplicon-based sequencing**

| **Sample** | **Mutation type** | **REFSEQ** | **Nucleotide change** | **Amino acid Transcript** | **whole tumor** | | **PD1+** | | **CD20+** | |
| --- | --- | --- | --- | --- | --- | --- | --- | --- | --- | --- |
|  |  |  |  |  | **Sanger** | **(%)** | **Sanger** | **(%)** | **Sanger** | **(%)** |
| PTCL2 | Splice site | NM_175629 | c.2409-1G>A | p.V690splice | positive | 30.6 | positive | 22.2 | positive | 41.5 |
| PTCL8 | Missense | NM_175629 | c.G2207A | p.R736H | positive | 5.6 | positive | 27.9 | negative | 0 |
| PTCL60 | Missense | NM_175629 | c.T2251G | p.F751V | positive | 11.5 | positive | 48.6 | positive | 6.2 |
| PTCL70 | Missense | NM_175629 | c.G2207A | p.R736H | positive | 35.7 | positive | 14.7 | negative | 0 |
| PTCL74 | Splice site | NM_175629 | c.2478+1G>- | p.L713splice | positive | 2.4 | positive | 27.9 | negative | 0 |
| PTCL127 | Missense | NM_153759 | c.G2078A | p.R693H | positive | 32 | positive | 55.7 | positive | 25.4 |
| PTCL136 | Missense | NM_153759 | c.G1175C | p.W392S | positive | 41.3 | positive | 56.9 | positive | 15.7 |

**Supplemental Table 10: Mutation profiles including *TET2/DNMT3A/RHOA/IDH2* mutations of *NOTCH1* mutated samples**

|  |  | **Allele frequencies (%)** | | | |
| --- | --- | --- | --- | --- | --- |
|  |  | **PTCL63** | | **PTCL74** | **PTCL78** |
| ***NOTCH1*** | **whole tumor** | **17.4** | | **22.4** | **23.7** |
|  | **PD1+** | **0** | | **0** | **0** |
|  | **CD20+** | **100** | | **ND** | **40.3** |
| ***TET2*** | **whole tumor** | **17.1** | **21** | **0** | **54.5** |
|  | **PD1+** | **29.1** | **60.1** | **0** | **79.7** |
|  | **CD20+** | **11.7** | **45.1** | **0** | **0** |
| ***DNMT3A*** | **whole tumor** | **0** | | **2.4** | **0** |
|  | **PD1+** | **0** | | **27.9** | **0** |
|  | **CD20+** | **0** | | **0** | **0** |
| ***RHOA*** | **whole tumor** | **13.8** | | **0** | **39.4** |
|  | **PD1+** | **16.9** | | **0** | **41.1** |
|  | **CD20+** | **0** | | **0** | **0** |
| ***IDH2*** | **whole tumor** | **12.2** | | **0** | **0** |
|  | **PD1+** | **15.6** | | **0** | **0** |
|  | **CD20+** | **0** | | **0** | **0** |

**Supplementary references**

1. Sakata-Yanagimoto M, Enami T, Yoshida K, Shiraishi Y, Ishii R, Miyake Y *et al.* Somatic RHOA mutation in angioimmunoblastic T cell lymphoma. *Nat Genet* 2014; 46(2): 171-175.
